# Association between smoking and the risk of dental implant failure in Korean adults: a nationwide cohort study

**DOI:** 10.4178/epih.e2026002

**Published:** 2026-01-14

**Authors:** Yu-Rin Kim, Minkook Son, Hyeon Ji Kim, Seon-Rye Kim

**Affiliations:** 1 Department of Dental Hygiene, Silla University, Busan, Korea; 2 Department of Physiology, Dong-A University College of Medicine, Busan, Korea; 3 Department of Data Sciences Convergence, Dong-A University, Busan, Korea; 4 Research Institute of Health Medical Education Convergence, Kangwon National University, Samcheok, Korea

**Keywords:** Dental implants, Dental restoration failure, Smoking, Dose–response relationship, Cohort studies, Korea

## Abstract

**OBJECTIVES:**

We evaluated the associations of smoking status, intensity, duration, and cumulative exposure with the risk of dental implant failure in Korean adults.

**METHODS:**

This retrospective cohort study utilized the National Health Insurance Service–Health Screening Cohort (2016–2019). Overall, 23,573 individuals who had completed the dental implant process were included. Smoking status was categorized as non-smoker, ex-smoker, or current smoker. Smoking intensity, duration, and cumulative exposure were classified using standardized thresholds (>10 cigarettes per day, >10 years, and >10 pack-years). Implant failure was defined as reimplantation or removal. Cox proportional hazards models were applied to estimate hazard ratios (HRs) and 95% confidence intervals (CIs), adjusting for demographic, clinical, and behavioral covariates.

**RESULTS:**

During follow-up, 605 implant failures occurred: 344 in non-smokers, 182 in ex-smokers, and 79 in current smokers. The corresponding incidence rates per 1,000 person-years were 11.56, 16.54, and 22.33, respectively. Current smoking was significantly associated with higher implant failure risk (adjusted HR, 1.59; 95% CI, 1.20 to 2.09), while ex-smokers displayed a non-significant increase (adjusted HR, 1.16; 95% CI, 0.94 to 1.45). A dose–response relationship was observed: smoking more than 10 cigarettes per day, smoking for more than 10 years, or exceeding 10 pack-years was associated with significantly increased risk.

**CONCLUSIONS:**

Smoking is a significant, dose–dependent risk factor for dental implant failure in Korean adults. Current smokers have the highest risk; smoking cessation may reduce adverse outcomes. These findings emphasize the importance of detailed smoking assessments and cessation counseling in implant care and public health strategies.

## GRAPHICAL ABSTRACT

[Fig f4-epih-48-e2026002]

## Key Message

This study examined differences in implant failure risk according to smoking status (current, former, never) and smoking intensity, duration, and cumulative exposure among adults who underwent dental implant placement. Compared with neversmokers, the risk of implant failure was significantly higher in current smokers, those who smoked ≥10 cigarettes/day, longterm smokers (≥10 years), and individuals with ≥10 pack-years of cumulative smoking exposure. Notably, the adverse impact of smoking on implant failure was greater in females than in males. In contrast, former smoking was not statistically associated with implant failure. These findings underscore the need for structured smoking-cessation counseling to improve oral health and optimize implant prognosis.

## INTRODUCTION

Dental implants are widely recognized as a reliable and effective treatment option for replacing missing teeth, offering superior functional and aesthetic outcomes compared with conventional prostheses. Implant-supported restorations reduce masticatory discomfort and improve quality of life among edentulous and partially edentulous patients [1]. With continuous advancements in surgical techniques, implant designs, and biomaterials, long-term survival rates now exceed 90% in most populations, establishing implants as a predictable solution in restorative dentistry [2-5].

Despite these achievements, implant success is not universal. Failure may occur early due to insufficient osseointegration or surgical trauma, or later as a result of peri-implantitis, marginal bone loss, or mechanical complications [6]. Beyond procedural and material factors, patient-related characteristics—such as systemic diseases, bone metabolism, and lifestyle behaviors—are decisive in determining implant prognosis [7-11]. Among these, smoking has consistently been identified as one of the most detrimental risk factors. Notably, growing evidence suggests that smoking cessation may attenuate the harmful effects of tobacco on systemic and oral health outcomes [7-11]. Former smokers have been reported to exhibit improved inflammatory profiles, enhanced tissue healing, and partial recovery of bone metabolism compared with current smokers, indicating that the biological impact of smoking may be at least partially reversible [10]. However, the extent to which smoking cessation mitigates the risk of dental implant failure remains insufficiently explored, particularly in large population-based studies. Clarifying implant prognosis among former smokers is therefore essential to strengthen the clinical rationale for smoking cessation and to inform evidence-based patient counseling. Tobacco use impairs angiogenesis, suppresses osteoblast and fibroblast activity, and disrupts bone remodeling, leading to delayed osseointegration and increased marginal bone loss [12-14]. Consequently, smokers experience higher rates of tooth loss, peri-implantitis, and implant failure than non-smokers [15].

Although the harmful effects of smoking are well established, the extent to which risk varies by smoking intensity, duration, and cumulative exposure remains unclear. While some studies have demonstrated a dose–response relationship—indicating that heavier or long-term smokers have a greater risk of implant failure [16]—others have reported inconsistent or non-significant associations [17]. This uncertainty highlights the need for large-scale, population-based investigations to clarify the quantitative relationship between smoking exposure and implant prognosis.

In Korea, the number of dental implant procedures has increased sharply since the expansion of National Health Insurance coverage for adults aged 65 years and older in 2016 [18]. This rapid growth in implant utilization underscores the public health importance of identifying modifiable behavioral risk factors, particularly smoking, that may compromise implant outcomes. However, no previous nationwide cohort study has comprehensively examined the effect of smoking exposure—including intensity, duration, and pack-years—on implant failure in the Korean population.

To address this knowledge gap, we conducted a large-scale, population-based cohort study using standardized health screening data to investigate the association between smoking and dental implant failure in Korean adults. Specifically, we aimed to evaluate the risk of implant failure according to smoking status (non-smoker, ex-smoker, or current smoker); determine whether a dose–response relationship exists across smoking intensity, duration, and cumulative exposure; and explore whether smoking cessation mitigates the risk of implant loss. The findings of this study are expected to provide robust epidemiologic evidence to guide clinical decision-making, improve patient counseling, and inform public health strategies for implant care in aging populations.

## MATERIALS AND METHODS

### Study population

**Figure 1.**
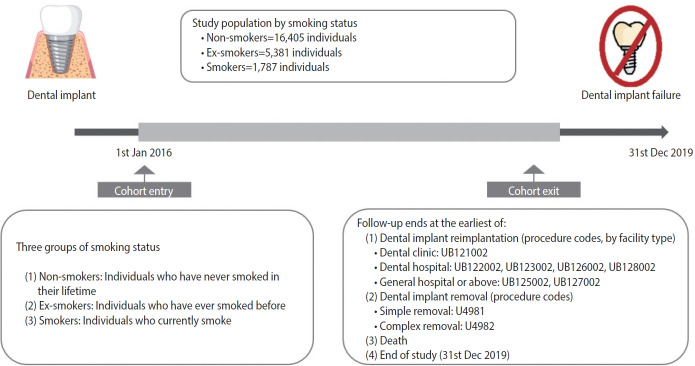
Flow diagram of the study.

This population-based retrospective cohort study used data from the Korea National Health Insurance Service–Health Screening Cohort (KNHIS-HEALS) from January 1, 2016, to December 31, 2019. The database includes de-identified information on demographics, diagnoses, prescribed medications, clinical procedures, and medical claims. The study population consisted of individuals who underwent dental implantation on or after January 1, 2016; the date of implantation was considered the index date. Records prior to 2016 were used solely to verify eligibility, ascertain medical history, and derive baseline covariates. Dental implant procedures were identified using the International Classification of Diseases, 10th revision (ICD-10) code K081 in combination with prespecified procedure codes (Supplementary Material 1). Because smoking was the primary exposure of interest, the analytic sample was restricted to participants with available health examination data within 12 months of the index date to minimize misclassification. Smoking status was self-reported during health examinations and categorized as non-smoker, ex-smoker, or current smoker. Smoking intensity, duration, and cumulative exposure were further classified using standardized thresholds: intensity >10 cigarettes per day, duration >10 years, and cumulative exposure >10 pack-years. The final analytic cohort included 23,573 adults who had completed the implant process. Participants were followed from the index date until implant failure, death, or December 31, 2019, whichever occurred first (Figure 1).

### Outcome

Dental implant failure was defined using specific procedure codes, listed in Supplementary Material 1. Two categories of failure were identified: reimplantation and removal surgery. Reimplantation was defined as procedures performed due to osseointegration failure among reimbursable implant recipients. Removal surgery was defined as procedures requiring removal of the implant fixture. Simple removal referred to fixture extraction due to osseointegration failure, whereas complex removal referred to cases requiring a trephine bur or removal kit, typically performed for fixture fracture or potential nerve injury. Reimplantation applied only to reimbursable implant recipients, whereas removal surgery was identified among both reimbursable and non-reimbursable cases.

### Covariates

Baseline characteristics were assessed at the time of the first implantation. Demographic variables included sex, age, income level (quartile), disability status, and residential area. Comorbidities were identified using ICD-10 codes, including hypertension (I10–I11), diabetes mellitus (E10–E14), dyslipidemia (E78), and osteoporosis (M81). The Charlson comorbidity index (CCI) was calculated based on diagnoses recorded within 1 year before implantation. Health screening data included body mass index (BMI), systolic blood pressure and diastolic blood pressure, fasting blood glucose, alcohol consumption, and physical activity. These variables were selected based on the prior literature indicating potential associations with implant prognosis.

### Statistical analysis

Baseline characteristics were compared across smoking status groups using analysis of variance for continuous variables and chi-square tests for categorical variables. The incidence of implant failure according to smoking status, daily cigarette consumption, smoking duration, and cumulative exposure (pack-years) was calculated per 1,000 person-years. Kaplan–Meier survival curves with log-rank tests were used to compare implant survival across smoking status groups. Cox proportional hazards regression models were utilized to estimate hazard ratios (HRs) and 95% confidence intervals (CIs) for implant failure by smoking status, intensity, duration, and cumulative exposure. The proportional hazards assumption was assessed with Schoenfeld residuals and log-cumulative hazard plots, and no violations were detected. Multivariable models were adjusted for demographic variables, comorbidities, CCI, BMI, alcohol consumption, and physical activity. A subgroup analysis by sex was conducted. All statistical analyses were performed using R version 4.3.0 (R Foundation for Statistical Computing, Vienna, Austria) and SAS version 8.3 (SAS Institute Inc., Cary, NC, USA). A two-sided p-value of less than 0.05 was considered to indicate statistical significance.

### Ethics statement

The study protocol was approved by the Institutional Review Board of Youngsan University (IRB No. YSUIRB-202412-HR-168-02) and conducted in accordance with the Declaration of Helsinki. Because the KNHIS-HEALS is an anonymized public dataset, the requirement for informed consent was waived. This study followed the Strengthening the Reporting of Observational Studies in Epidemiology (STROBE) guidelines.

## RESULTS

### Baseline characteristics by smoking status

A total of 23,573 individuals who completed dental implantation were included in the final analysis (Figure 1). Of these, 16,405 were non-smokers, 5,381 were ex-smokers, and 1,787 were current smokers. Baseline characteristics by smoking status are summarized in Table 1. Compared with non-smokers, both ex-smokers and current smokers were more likely to be male and younger and had a higher prevalence of diabetes but a lower prevalence of hypertension, dyslipidemia, and osteoporosis (p<0.001). Non-smokers had a higher mean BMI, systolic blood pressure, and diastolic blood pressure than current smokers, but lower fasting blood glucose levels. Mean pack-years were higher among current smokers than among ex-smokers. Alcohol consumption was more prevalent among current smokers and ex-smokers than among non-smokers, whereas regular exercise was less frequent among current smokers. Implant failure occurred in 344 non-smokers, 182 ex-smokers, and 79 current smokers.

### Association between smoking and dental implant failure

**Figure 2.**
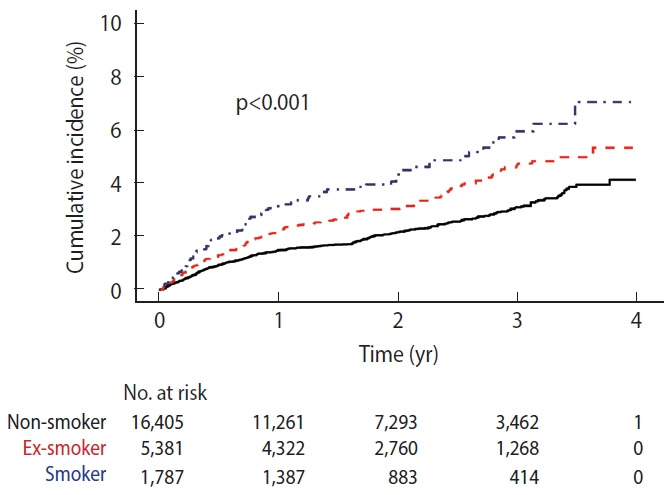
Kaplan–Meier curve for dental implant failure according to smoking status.

During follow-up, 605 implant failures were observed: 344 among non-smokers (2.1%), 182 among ex-smokers (3.4%), and 79 among current smokers (4.4%) (p<0.001) (Table 1). The crude incidence rates per 1,000 person-years were 11.56 for non-smokers, 16.54 for ex-smokers, and 22.33 for current smokers. In the multivariable Cox proportional hazards model adjusted for demographic, clinical, and behavioral factors, current smoking was significantly associated with an increased risk of implant failure (adjusted HR, 1.59; 95% CI, 1.20 to 2.09), whereas ex-smokers showed a non-significant elevation in risk (adjusted HR, 1.16; 95% CI, 0.94 to 1.45) (Table 2). Kaplan–Meier curves demonstrated significantly lower implant survival among current smokers compared with non-smokers (log-rank p<0.001) (Figure 2).

Subgroup analyses suggested a dose–response relationship. Patients who smoked more than 10 cigarettes per day had a significantly higher risk of implant failure (adjusted HR, 1.31; 95% CI, 1.06 to 1.61) than non-smokers, whereas those who smoked 10 or fewer cigarettes per day did not differ significantly (adjusted HR, 1.07; 95% CI, 0.74 to 1.53). Similarly, individuals with more than 10 years of smoking history faced an increased risk (adjusted HR, 1.27; 95% CI, 1.04 to 1.57), while shorter durations did not display statistical significance. In the analysis by cumulative exposure, patients with more than 10 pack-years had a higher risk of implant failure (adjusted HR, 1.30; 95% CI, 1.05 to 1.62), whereas those with fewer than 10 pack-years did not differ significantly from non-smokers (Table 2).

### Subgroup analyses according to sex

**Figure 3.**
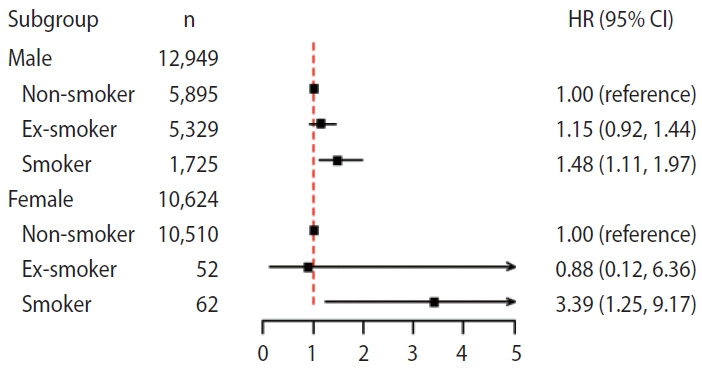
Subgroup analysis according to sex. HR, hazard ratio; CI, confidence interval.

Sex-stratified analyses revealed consistent positive associations between smoking and implant failure in both males and females. The adjusted HRs for implant failure among ex-smokers were not statistically significant in either sex. Among current smokers, the adjusted HR was 1.48 (95% CI, 1.11 to 1.97) in males and 3.39 (95% CI, 1.25 to 9.17) in females, indicating a more pronounced association among females (Figure 3).

## DISCUSSION

In this large nationwide population-based cohort of Korean adults, current smoking was found to be an independent and significant risk factor for dental implant failure. After adjustment for demographic, clinical, and lifestyle variables, current smokers had a markedly higher risk of implant failure than non-smokers, whereas ex-smokers showed a modest but non-significant elevation in risk. Moreover, a clear dose–response pattern was observed: greater daily cigarette consumption, longer smoking duration, and higher cumulative exposure (pack-years) were each associated with a progressively increased risk of implant failure. To our knowledge, 4this is one of the largest Korean cohort studies to comprehensively quantify the influence of smoking exposure on implant survival.

Our findings corroborate prior evidence identifying smoking as a major determinant of implant prognosis [19,20]. Meta-analyses consistently demonstrate that smokers face significantly higher rates of implant failure than non-smokers [15,21-23], and systemic or behavioral factors such as smoking have been emphasized as crucial prognostic indicators [24]. Biologically, nicotine and other toxic constituents of tobacco smoke impair angiogenesis, suppress osteoblast and fibroblast activity, and disrupt bone remodeling, thereby compromising osseointegration and predisposing individuals to peri-implantitis [12,13]. Consequently, smokers tend to experience greater marginal bone loss around implants than non-smokers [14]. These biological mechanisms provide a plausible basis for the adverse outcomes observed in this study. Our findings may also be interpreted in light of established pathophysiological mechanisms. Smoking-induced impairment of vascularization and delayed wound healing may hinder early implant stabilization, whereas persistent immune dysregulation and chronic inflammatory responses increase vulnerability to peri-implant tissue breakdown over time. Moreover, the observed dose–response relationship is biologically plausible, as greater smoking intensity and cumulative exposure are likely to amplify hypoxic stress, inflammatory burden, and disruption of bone remodeling processes that are critical for osseointegration. In contrast, the attenuated risk observed among former smokers suggests that partial recovery of vascular function, immune competence, and bone metabolism may occur after smoking cessation, thereby improving implant prognosis. Integrating these mechanistic insights with our epidemiologic findings strengthens the biological coherence of the observed associations and supports smoking exposure as a key modifiable determinant of dental implant outcomes.

Interestingly, sex-stratified analyses showed that the adverse impact of smoking on implant failure was more pronounced in females than in males, despite most female participants being non-smokers. While Mohajeri Tehrani et al. [25] reported a higher risk of implant failure in males, other studies found no significant sex-related differences [26]. These discrepancies may reflect differences in smoking prevalence, biological susceptibility, or hormonal influences, which warrant further investigation.

A key contribution of this study is the confirmation of a dose–response relationship between smoking exposure and implant failure. Participants who smoked more than 10 cigarettes per day had a significantly elevated risk, whereas light smokers (≤10 cigarettes/day) did not show a statistically significant increase compared with non-smokers. This finding aligns with previous systematic reviews demonstrating that heavy smoking markedly increases failure risk [27], although even low-intensity smoking may still exert detrimental oral effects [28]. Furthermore, long-term smokers (>10 years) or those with more than 10 pack-years of cumulative exposure had a substantially higher risk than non-smokers, underscoring the cumulative biological burden of tobacco on implant prognosis [10,29,30]. These results highlight the need for future research to treat smoking exposure as a continuous quantitative variable rather than a categorical trait.

Despite the robustness of this study, several limitations merit consideration. First, smoking status and exposure were self-reported, which may introduce recall bias or lead to underestimation of true consumption. Objective biomarkers such as serum or salivary cotinine levels could improve exposure assessment in future studies. Second, the NHIS-HEALS database lacks clinical information on implant-specific factors (e.g., bone quality, implant system, surgical technique, operator experience) that may influence outcomes. Third, although the follow-up period captured medium-term outcomes, longer observation is required to explore delayed or late implant failures. Finally, as an observational study, residual confounding cannot be completely excluded despite multivariable adjustment.

The clinical and public health implications of these findings are substantial. As dental implants become increasingly common in Korea—particularly since the expansion of National Health Insurance coverage for older adults in 2016—smoking gains importance as a modifiable behavioral risk factor that undermines treatment success. Comprehensive smoking assessment (including intensity, duration, and pack-years) should be integrated into pre-implant evaluation, as current smoking status alone may not capture heterogeneity in risk. Moreover, our finding that ex-smokers showed only a modest and non-significant increase in failure risk suggests that smoking cessation may partially reverse the adverse biological effects of tobacco, reinforcing the importance of cessation counseling before implant placement. From a clinical perspective, these findings support classifying former smokers as a distinct risk group rather than combining them with current smokers in implant risk assessment. The observed attenuation of failure risk among ex-smokers underscores smoking cessation as a meaningful intervention that may improve implant prognosis. From a public health standpoint, these results reinforce the role of dental professionals in providing smoking cessation counseling as part of comprehensive implant care, particularly in aging populations in which implant utilization is rapidly increasing. Emphasizing the potential benefits of cessation may increase patient motivation, optimize treatment outcomes, and reduce the long-term burden of implant-related complications. Integrating tobacco cessation strategies into dental care could enhance implant survival, improve oral health outcomes, and reduce the healthcare burden associated with implant-related complications.

This nationwide cohort study demonstrated that smoking is a significant, dose-dependent risk factor for dental implant failure among Korean adults. Current smokers had a markedly higher risk of implant failure, with failure risk increasing in proportion to smoking intensity, duration, and cumulative exposure. Although ex-smokers showed a modest, non-significant increase in risk, these findings suggest that smoking cessation may partially mitigate adverse outcomes. These results highlight the importance of incorporating comprehensive smoking assessments and cessation interventions into implant treatment planning, as well as integrating tobacco control strategies into broader public health initiatives, to improve oral health outcomes and implant longevity.

## Figures and Tables

**Figure f4-epih-48-e2026002:**
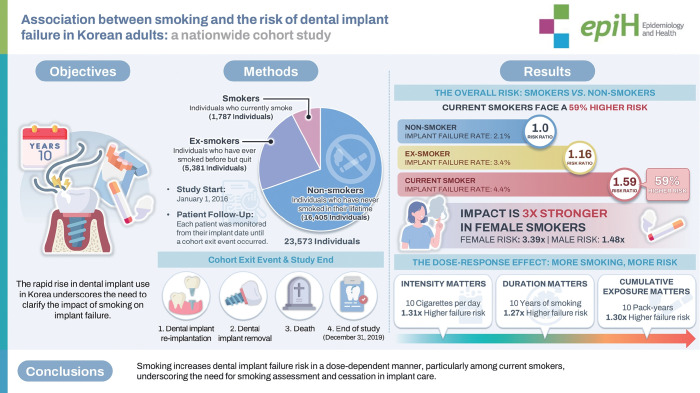


**Table 1.** Baseline characteristics of the study population

Characteristics		Non-smoker (n=16,405)	Ex-smoker (n=5,381)	Smoker (n=1,787)	p-value
Sex	Male	5,895 (35.9)	5,329 (99.0)	1,725 (96.5)	<0.001
	Female	10,510 (64.1)	52 (1.0)	62 (3.5)	
Age (yr)		71.5±4.9	70.6±4.5	69.4±3.9	<0.001
Income level (quartile)	1st	2,907 (17.7)	865 (16.1)	313 (17.5)	<0.001
	2nd	2,689 (16.4)	1,076 (20.0)	429 (24.0)	
	3rd	4,390 (26.8)	1,451 (27.0)	537 (30.1)	
	4th	6,419 (39.1)	1,989 (37.0)	508 (28.4)	
Disability	No	14,320 (87.3)	4,766 (88.6)	1,555 (87.0)	0.006
	Mild	305 (1.9)	110 (2.0)	47 (2.6)	
	Severe	1,780 (10.9)	505 (9.4)	185 (10.4)	
Residence	Rural	6,739 (41.1)	1,781 (33.1)	678 (37.9)	<0.001
	Urban	9,666 (58.9)	3,600 (66.9)	1,109 (62.1)	
Hypertension		11,456 (69.8)	3,729 (69.3)	1,161 (65.0)	<0.001
Diabetes		3,965 (24.2)	1,534 (28.5)	543 (30.4)	<0.001
Dyslipidemia		9,755 (59.5)	2,841 (52.8)	924 (51.7)	<0.001
Osteoporosis		2,547 (15.5)	334 (6.2)	120 (6.7)	<0.001
Charlson comorbidity index	0	3,495 (21.3)	1,217 (22.6)	451 (25.2)	0.007
	1	4,137 (25.2)	1,324 (24.6)	428 (24.0)	
	2	3,355 (20.5)	1,101 (20.5)	361 (20.2)	
	≥3	5,418 (33.0)	1,739 (32.3)	547 (30.6)	
Body mass index (kg/m²)		24.5±3.0	24.4±2.7	23.7±2.8	<0.001
Systolic blood pressure (mmHg)		128.7±14.6	127.9±13.9	126.4±14.5	<0.001
Diastolic blood pressure (mmHg)		76.3±9.4	76.3±9.3	75.7±9.4	0.017
Fasting blood glucose (mg/dL)		104.6±24.6	107.6±25.5	107.6±27.1	<0.001
Pack-years		0.0±0.0	21.0±18.0	25.7±16.3	<0.001
Alcohol consumption		3,639 (22.2)	3,104 (57.7)	1,095 (61.3)	<0.001
Regular exercise (times/wk)	No	9,368 (57.1)	2,637 (49.0)	1,044 (58.4)	<0.001
	1–2	2,079 (12.7)	1,011 (18.8)	302 (16.9)	
	3–4	2,282 (13.9)	880 (16.4)	205 (11.5)	
	≥5	2,676 (16.3)	853 (15.9)	236 (13.2)	
Dental implant failure		344 (2.1)	182 (3.4)	79 (4.4)	<0.001

Values are presented as number (%) or mean±standard deviation.

**Table 2.** Association between smoking and dental implant failure

Variables	Total (n)	Events (n)	Follow-up duration (PY)	Incidence rate (per 1,000 PY)	Crude	p-value	Adjusted^1^	p-value
Non-smoker	16,405	344	29,766.93	11.56	1.00 (reference)		1.00 (reference)	
Ex-smoker	5,381	182	11,004.10	16.54	1.45 (1.21, 1.74)	0.001	1.16 (0.94, 1.45)	0.172
Smoker	1,787	79	3,537.55	22.33	1.95 (1.53, 2.49)	<0.001	1.59 (1.20, 2.09)	0.001
Smoking amount (cigarettes/day)								
Non-smoker	16,405	344	29,766.93	11.56	1.00 (reference)		1.00 (reference)	
0–10	1,165	36	2,452.42	14.68	1.29 (0.92, 1.83)	0.140	1.07 (0.74, 1.53)	0.728
>10	6,003	225	12,089.23	18.61	1.63 (1.38, 1.93)	<0.001	1.31 (1.06, 1.61)	0.012
Smoking duration (yr)								
Non-smoker	16,405	344	29,766.93	11.56	1.00 (reference)		1.00 (reference)	
0–10	579	19	1,159.10	16.39	1.44 (0.90, 2.28)	0.125	1.17 (0.73, 1.89)	0.518
>10	6,589	242	13,382.55	18.08	1.59 (1.35, 1.87)	<0.001	1.27 (1.04, 1.57)	0.022
Pack-years^2^								
Non-smoker	16,405	344	29,766.93	11.56	1.00 (reference)		1.00 (reference)	
0–10	1,633	53	3,303.32	16.04	1.41 (1.05, 1.88)	0.021	1.14 (0.83, 1.56)	0.412
>10	5,535	208	11,238.33	18.51	1.62 (1.37, 1.93)	<0.001	1.30 (1.05, 1.62)	0.015

Values are presented as hazard ratio (95% confidence interval). PY, person-years.

1 Adjusted for sex, age, income level, disability, residence area, hypertension, diabetes, dyslipidemia, osteoporosis, Charlson comorbidity index, body mass index, alcohol consumption, and regular exercise.

2 20 cigarettes are equivalent to 1 pack.

## Supplementary materials

Supplementary Material 1. Dental implant procedure code
