# Supplementary material for: Association between smoking and the risk of dental implant failure in Korean adults: a nationwide cohort study
Source: Epidemiol Health. 2026 Jan 14;48:e2026002. doi: 10.4178/epih.e2026002 (PMC12946569; doi:10.4178/epih.e2026002)
Supplement: Supplementary Material 1. — Dental implant procedure code [file epih-48-e2026002-Supplementary-1.docx]

Supplementary Material 1. Dental implant procedure code

|  | **ICD-10** | **Procedure content** | **Procedure codes** |
| --- | --- | --- | --- |
| **Dental implant** | K081 | Stage 1  Diagnosis and treatment plan | UB111, UB112, UB113,  UB115, UB116, UB117, UB118 |
|  |  | Stage 2  Dental implant fixture (body) placement surgery | UB121, UB122, UB123  UB125, UB126, UB127, UB128 |
|  |  | Stage 3  Dental implant prosthesis restoration | UB131, UB132, UB133,  UB135, UB136, UB137, UB138 |
| **Dental implant failure** |  | Definition 1  Dental Implant reimplantation  (only reimbursable dental implants) | UB121002, UB122002, UB123002 UB125002, UB126002,  UB127002, UB128002 |
|  |  | Definition 2  Dental implant removal surgery  (includes both reimbursable and non-reimbursable dental implants) | Simple removal: U4981 |
|  |  |  | Complex removal: U4982 |
